# Sirtuin Inhibition Adversely Affects Porcine Oocyte Meiosis

**DOI:** 10.1371/journal.pone.0132941

**Published:** 2015-07-15

**Authors:** Liang Zhang, Rujun Ma, Jin Hu, Xiaolin Ding, Yinxue Xu

**Affiliations:** 1 College of Animal Science & Technology, Nanjing Agricultural University, Nanjing, China; 2 College of Veterinary Medicine, Nanjing Agricultural University, Nanjing, China; China Agricultural University, CHINA

## Abstract

Sirtuins have been implicated in diverse biological processes, including oxidative stress, energy metabolism, cell migration, and aging. Here, we employed Sirtuin inhibitors, nicotinamide (NAM) and Sirtinol, to investigate their effects on porcine oocyte maturation respectively. The rate of polar body extrusion in porcine oocytes decreased after treatment with NAM and Sirtinol, accompanied with the failure of cumulus cell expansion. We further found that NAM and Sirtinol significantly disrupted oocyte polarity, and inhibited the formation of actin cap and cortical granule-free domain (CGFD). Moreover, the abnormal spindles and misaligned chromosomes were readily detected during porcine oocyte maturation after treatment with NAM and Sirtinol. Together, these results suggest that Sirtuins are involved in cortical polarity and spindle organization in porcine oocytes.

## Introduction

Maturation of mammalian oocytes is characterized by breakdown of the germinal vesicle (GV), followed by chromatin condensation and reorganization of microtubules during first meiosis (MI). Subsequently, the spindle migrates along its long axis toward the oocyte cortex, and emission of the first polar body occurs in the metaphase of second meiosis (MII). The fully matured oocytes are arrested at metaphase II until fertilization [1]. Oocyte maturation involves two aspects, cytoplasmic and nuclear maturation. Both steps are essential for the formation of a fertilizable egg that can subsequently develop into a live offspring. Nuclear maturation primarily includes chromosomal condensation and segregation. Cytoplasmic maturation refers to the processes that prepare the egg for activation and preimplantation development [2]. Actin is important for mammalian oocyte maturation. Actin cytoskeleton is involved in asymmetric spindle positioning and cortical polarization during meiotic division in all organisms from mice to humans [3–8]. Lenart et al. have demonstrated that chromosome congression is driven by the actin network [9], and the actin-enriched cortical domain promotes the extrusion of the first polar body during myosin assembly [10–12]. Moreover, the distribution of cortical granules (CGs) during oocyte maturation has been used as a critical criterion to assess cytoplasmic maturation. CGs of mature oocytes migrate to the cortex to form a continuous layer overlying the oolemma [13–16]. In summary, oocyte maturation is a complex biological process, and development of oocyte polarity involves the assembly of actin and CGs.

Sirtuins are nicotinamide adenine dinucleotide (NAD)-dependent deacetylases that are highly conserved from bacteria to humans. The Sirtuin family comprises seven proteins (Sirt1–-Sirt7) in mammals, all of which have different tissue specificities, subcellular localization, enzymatic activities, and targets [17]. Numerous studies have shown that Sirtuins deacetylate histone H3/H4 or non-histone substrates such as Peroxisome proliferator-activated receptor-gamma coactivator (PGC-1α) and Liver X receptor (LXR) α to participate in transcriptional regulation, chromatin modification, and cell migration [18–20]. NAM and Sirtinol have been reported to affect embryo development [21, 22]. A previous study has demonstrated that Sirt2 regulates actin assembly and spindle organization in mouse oocyte during meiosis [23].

Although Sirtuins participate in multiple critical biological processes, to our knowledge, the precise roles of Sirtuins in porcine oocytes during meiosis have not been elucidated. In this study, we investigated the effects of inhibition of Sirtuin activity on porcine oocyte maturation using two specific inhibitors, NAM and Sirtinol. Treatment with these inhibitors reduced the rate of polar body extrusion and disrupted actin distribution and formation of cortical granule-free domain (CGFD). Moreover, inhibition of Sirtuin activity led to spindle defects and chromosome misalignment in porcine oocytes.

## Materials and Methods

### Ethics statement

Animals use and care were in accordance with Animal Research Institute Committee guidelines prescribed by Nanjing Agricultural University, China. Ovaries were obtained from 6 month-old Duroc gilts at the Nanjing Tianhuan Food Corporation slaughterhouse in china: Nanjing (NJ), Jiangsu Province (31°52′55″N,118°40′20″E) and transported to the laboratory within 3 h after death in sterile physiological saline (0.9% natrium chloride) containing 75 mg/L of penicillin and 50 mg/L streptomycin at 30–35°C. This study was specifically approved by the Committee of Animal Research Institute, Nanjing Agricultural University, China, and permission was obtained from the Nanjing Tianhuan Food Corporation slaughterhouse to use the ovaries.

### Antibodies and chemicals

Mouse monoclonal anti-α-tubulin antibody (F2168), NAM, and Sirtinol were purchased from Sigma (St. Louis, MO, USA); fluorescein isothiocyanate (FITC)-conjugated goat anti-rabbit immunoglobulin (IgG) and tetramethylrhodamine (TRITC)-conjugated goat anti-rabbit IgG were purchased from Thermo Fisher Scientific Inc. (Rockford, IL, USA). Other chemicals and culture media were purchased from Sigma (St. Louis, MO, USA) unless stated otherwise.

### Porcine oocyte isolation and culture

Porcine ovaries were collected from prepubertal gilts at a local slaughterhouse and were transported to the laboratory in sterile saline (0.9% NaCl) containing 75 mg/mL penicillin G and 50 mg/mL streptomycin sulfate maintained at 37°C. Cumulus-oocyte complexes (COCs) were aspirated from medium-sized follicles (3–6 mm in diameter) from the ovaries with a 20-gauge needle and a syringe. COCs surrounded by a compact cumulus mass and with uniform ooplasm were isolated from the cellular debris. The COCs were rinsed 4 times in a maturation medium containing tissue culture medium (TCM)-199 supplemented with 0.1% polyvinyl alcohol, 0.57 mM cysteine, 0.91 mM sodium pyruvate, 3.05 mM glucose, 75 mg/L penicillin, 50 mg/L streptomycin, 10% (v/v) pig follicular fluid (pFF), 10 ng/mL epidermal growth factor (EGF), 10 IU/mL pregnant mare's serum gonadotropin (PMSG), 10 IU/mL human chorionic gonadotropin (hCG). Ninety oocytes were cultured in 500 μL of maturation medium in a 4-well dish (NUNC) at 38.5°C in an atmosphere of 5% CO_2_ and saturated humidity.

For oocyte maturation, the COCs were cultured for 44 h. After culturing, the COCs were treated with 0.02% (w/v) hyaluronidase (in TCM-199) for 5 min at 38°C. The oocytes were then separated from the surrounding cumulus cells completely by pipetting gently with a fine-bore pipette. After 4 rinses, the cumulus-free oocytes were used for further analysis. To calculate the maturation rate, cumulus-free porcine oocytes were gathered together in a 100-μL TCM199 droplet, and then each oocyte was rotated several times one by one under a Nikon Diaphot ECLIPSE TE300 inverted microscope (Nikon UK. Ltd) to observe polar body extrusion. Oocytes with clearly extruded polar bodies were considered mature.

### Treatment of porcine oocytes with NAM and Sirtinol *in vitro*

Sirtuin inhibitor, NAM and Sirtinol were employed to inhibit the activity of intracellular Sirtuins during porcine oocytes meiosis. NAM and Sirtinol were diluted to 5M and 1 M stock solutions in dimethyl sulfoxide (DMSO), respectively, and stored at −20°C. For treatment, the stock solution was diluted with TCM-199 to attain final concentrations of 5 mM NAM and 100 μM Sirtinol. Oocytes were cultured in maturation medium containing NAM and Sirtinol respectively for 44 h to obtain MII oocytes, then oocytes were washed 3 times and stained for immunofluorescence assays. Control oocytes were treated with the same concentration of DMSO before examination. The GV oocytes were incubated in maturation medium containing 5 mM NAM and 100 μM Sirtinol for 26 h to obtain the MI stage oocytes for the examination of actin assembly, CGFD and spindle organization.

### Immunofluorescence staining and confocal microscopy

Oocytes were fixed in 4% paraformaldehyde (in phosphate-buffered saline [PBS]) at room temperature for 1 h, and then permeabilized with 0.5% Triton X-100 in PBS for 20 min. Then, the oocytes were transferred into a blocking buffer (1% bovine serum albumin [BSA]-supplemented PBS) for 1 h at room temperature. For α-Tubulin-FITC staining, the oocytes were incubated with anti-α-Tubulin-FITC antibody (1:200 dilution) at 4°C overnight. For Actin staining, the oocytes were incubated with phalloidin-TRITC (5 μg/mL in PBS) at room temperature for 1 h. After 3 washes (1 min each) with a wash buffer, the oocytes were incubated with Hoechst 33342 at room temperature for 10 min. After staining, the oocytes were mounted on glass slides and observed with a confocal laser-scanning microscope (Zeiss LSM 18 710 META, Germany).

### Fluorescence intensity analysis

Actin staining intensity was analyzed using the Image J software. Control- and treatment-group oocytes were mounted on the same glass slide. After immunofluorescence staining, the average fluorescence intensity per unit area within a region of interest (ROI) of the immunofluorescence images was determined. Finally, the average values of all measurements were used to compare the final average intensities between the control and treatment groups. At least 3 replicates were used for each group, and iterations containing 30 oocytes were examined in each group.

### Statistical analysis

At least three replicates were done for each treatment used. Results are presented as means ± standard error of the mean (SEM). Statistical comparisons were made by analysis of variance (ANOVA), followed by Duncan’s multiple comparisons test. A *p*-value of <0.05 was considered significant.

## Results

### Effects of NAM and Sirtinol on meiotic maturation of porcine oocytes

**Fig 1:**
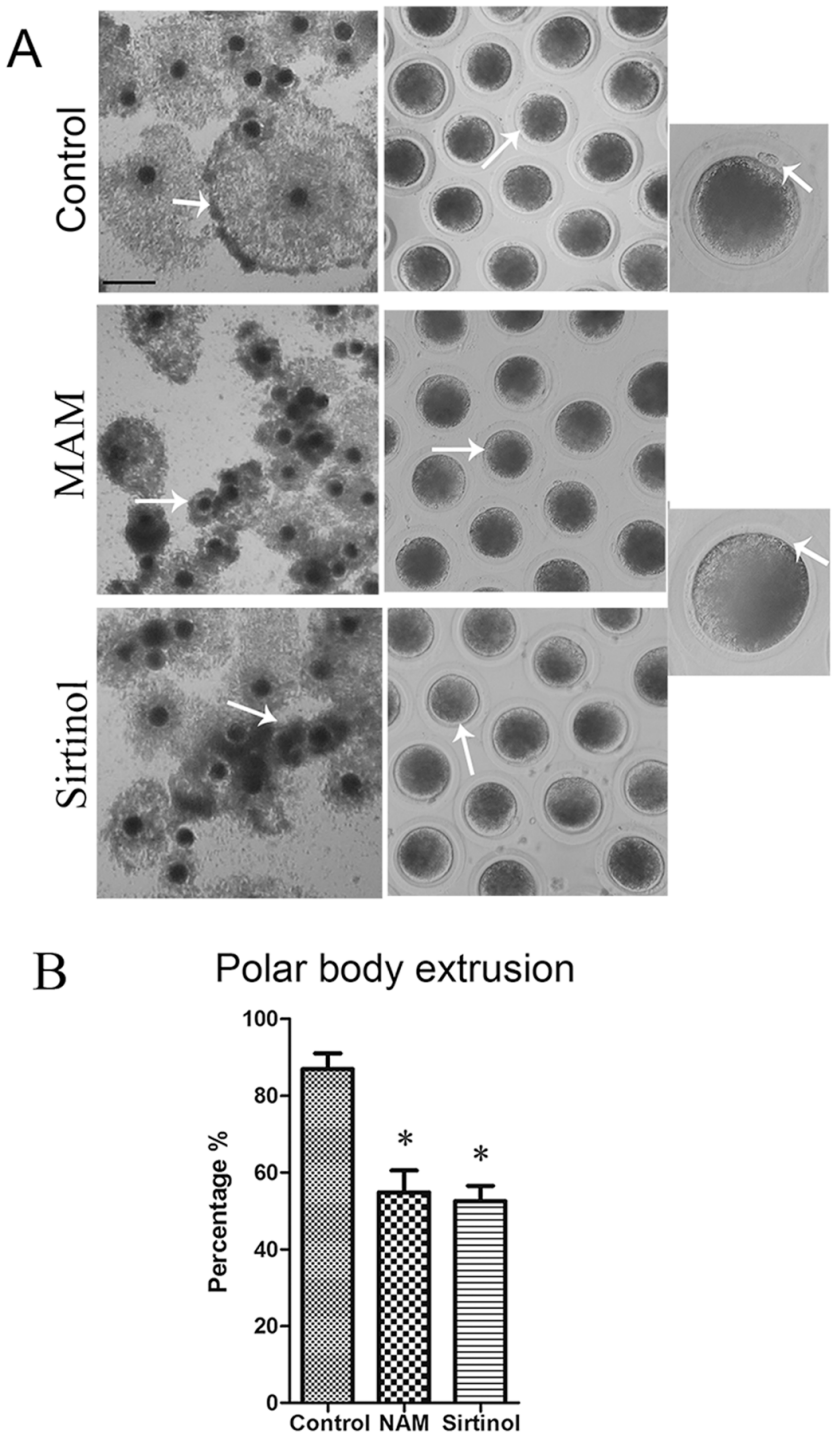
Treatment with nicotinamide (NAM) and Sirtinol affects meiotic maturation in porcine oocytes. (A) In cumulus-oocyte complexes (COCs), the peripheral layer of the cumulus expanded to more than 5 layers in the control group, whereas it became progressively thinner in the NAM- and Sirtinol-treated COCs. Most oocytes in the control group extruded the first polar body (indicated by white arrow). Upon treatment with NAM and Sirtinol, a large proportion of oocytes failed to extrude polar bodies. Bar = 100 μm. (B) Rate of polar body extrusion in the control, NAM-, and Sirtinol-treated groups. Rate of polar body extrusion in treatment groups was significantly reduced comparing to control group. *, significant, *p* < 0.05.

To investigate the possible involvement of Sirtuins in porcine oocyte meiotic maturation, COCs were treated with two kinds of Sirtuin inhibitors: NAM and Sirtinol. As reported in a previous study [22], concentrations of 5 mM NAM and 100 μM Sirtinol were used in this study. The peripheral layer of the cumulus expanded to more than 5 layers in the control group. However, the cumulus cells did not expand as much in the NAM and Sirtinol-treated COCs (Fig 1A). Moreover, after 40 h of *in vitro*-culture, most of control oocytes extruded small polar bodies and were arrested at the MII stage, whereas only approximately 50% of the NAM and Sirtinol treated-oocytes extruded the first polar body, even after an additional culture period (86.9 ± 4.1%, n = 38 [control] vs. 54.9 ± 5.7%, n = 59 [NAM] vs. 52.6 ± 4.0%, n = 62 [Sirtinol]; *p* < 0.05; Fig 1B) (“n” is the average number of each experiment).

### NAM and Sirtinol treatment disrupts actin formation

**Fig 2:**
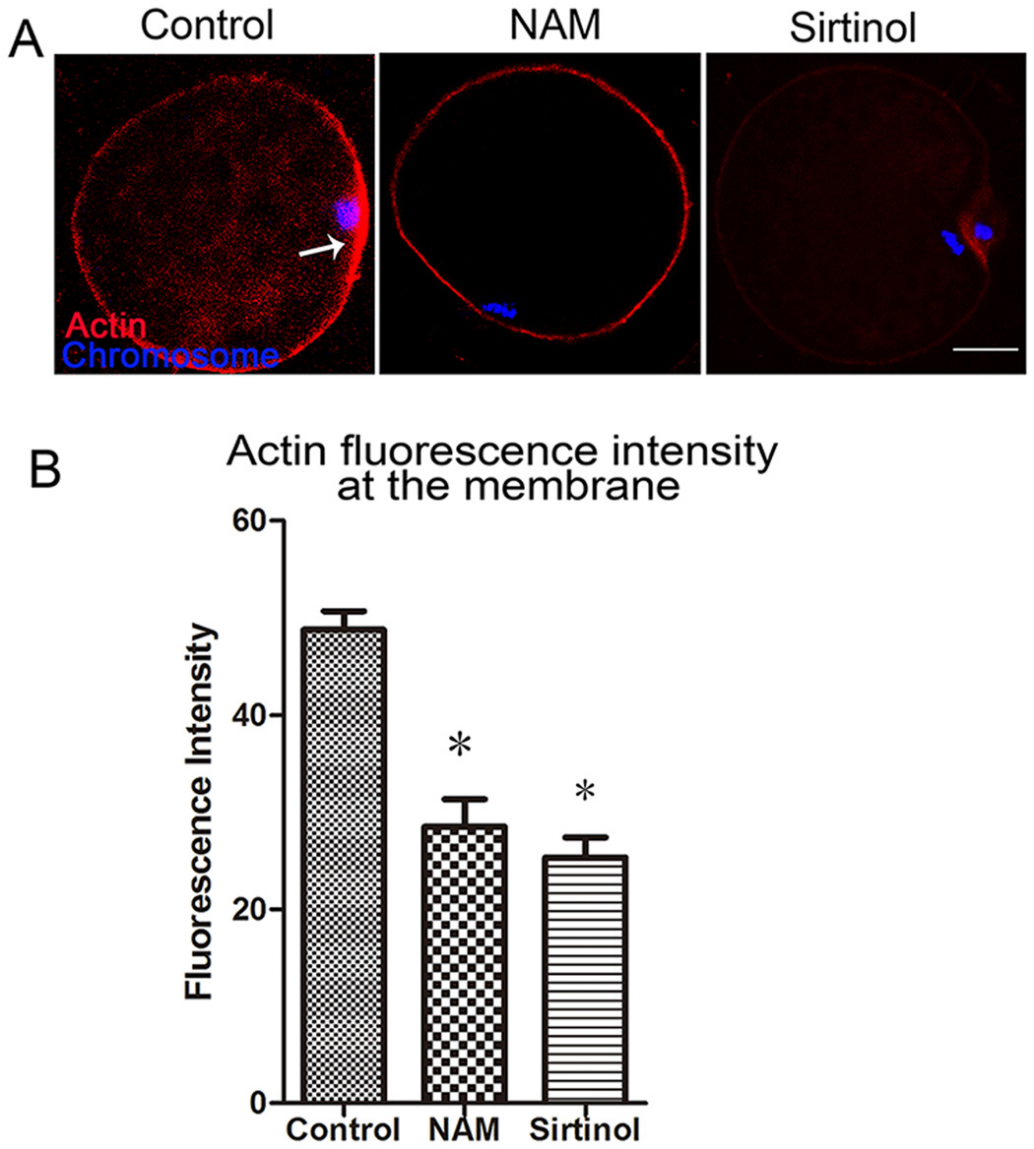
NAM and Sirtinol treatment disrupts actin cap formation. (A) Actin signals in the oocytes were examined by immunofluorescence staining. Oocytes treated with NAM and Sirtinol exhibited profoundly decreased actin fluorescence intensity. Bar = 20 μm (B) Actin fluorescence intensity at the membrane in porcine oocytes. The signal intensity of ROI of actin was the entire area of the oocyte membrane. Compared to control oocytes, actin fluorescence intensity was significantly decreased in inhibitor-treated oocytes. *significant, *p* < 0.05.

Polar body extrusion is an actin-based process, and hence, failure of polar body emission may be attributed to disruption of oocyte polarity. To confirm this hypothesis, we analyzed the oocytes by immunostaining against F-Actin. As shown in Fig 2A, an actin cap was developed in the cortex of control oocytes, while the formation of actin cap was significantly reduced in the NAM- and Sirtinol-treated oocytes. In addition, we examined the expression of actin at the cortex of control and treated oocytes. As shown in Fig 2B, the fluorescence intensity of actin at the membrane in the control oocytes was significantly higher than that in the NAM and Sirtinol-treated oocytes (48.8 ± 1.9, n = 45 [control] vs. 28.5 ± 2.8, n = 45 [NAM] vs. 25.3 ± 2.1, n = 45 [Sirtinol]; *p* < 0.05) (“n” is the average number of each experiment). Thus, these results suggest that NAM and Sirtinol affect actin assemblyand cap formation.

### NAM and Sirtinol treatment disrupts the formation of CGFD

**Fig 3:**
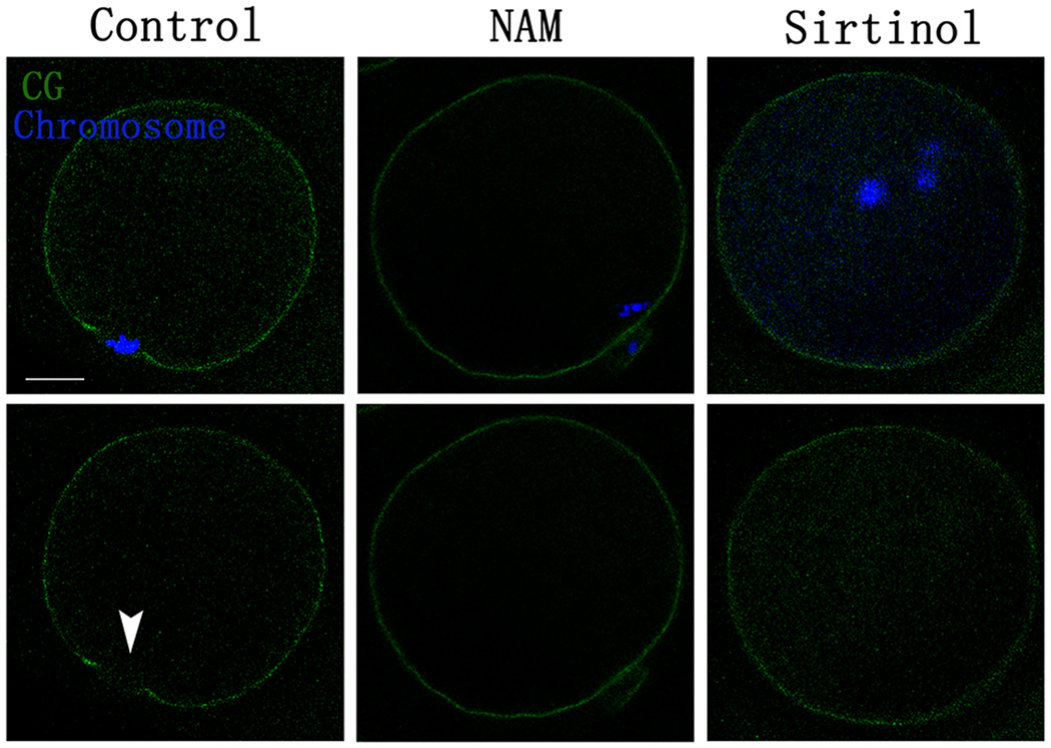
Effects of NAM and Sirtinol on cortical granule-free domain (CGFD) formation in porcine oocytes. In the control group, cortical granules (CGs) were absent at the cortex near the site of polar body extrusion in MI oocyte. However, in the treatment group, cortical granules were distributed uniformly across the entire cortex. CGFD formation was failed to observation. The arrow indicates a CGFD. Green, CGs; blue, chromatin. Bar = 20 μm.

Formation of CGFD is another important aspect of oocyte polarity. As shown in Fig 3, CGs were absent near the cortex region overlying the chromosomes in control MII oocytes. However, in NAM- and Sirtinol-treated oocytes, CGs were uniformly distributed throughout the entire cortex. This result suggests that CGFD formation was disrupted by NAM and Sirtinol in porcine oocytes. Together, the failure to form the actin cap and CGFD indicates defective oocyte polarity after NAM and Sirtinol treatment.

### NAM and Sirtinol treatment result in spindle defects in porcine oocytes

**Fig 4:**
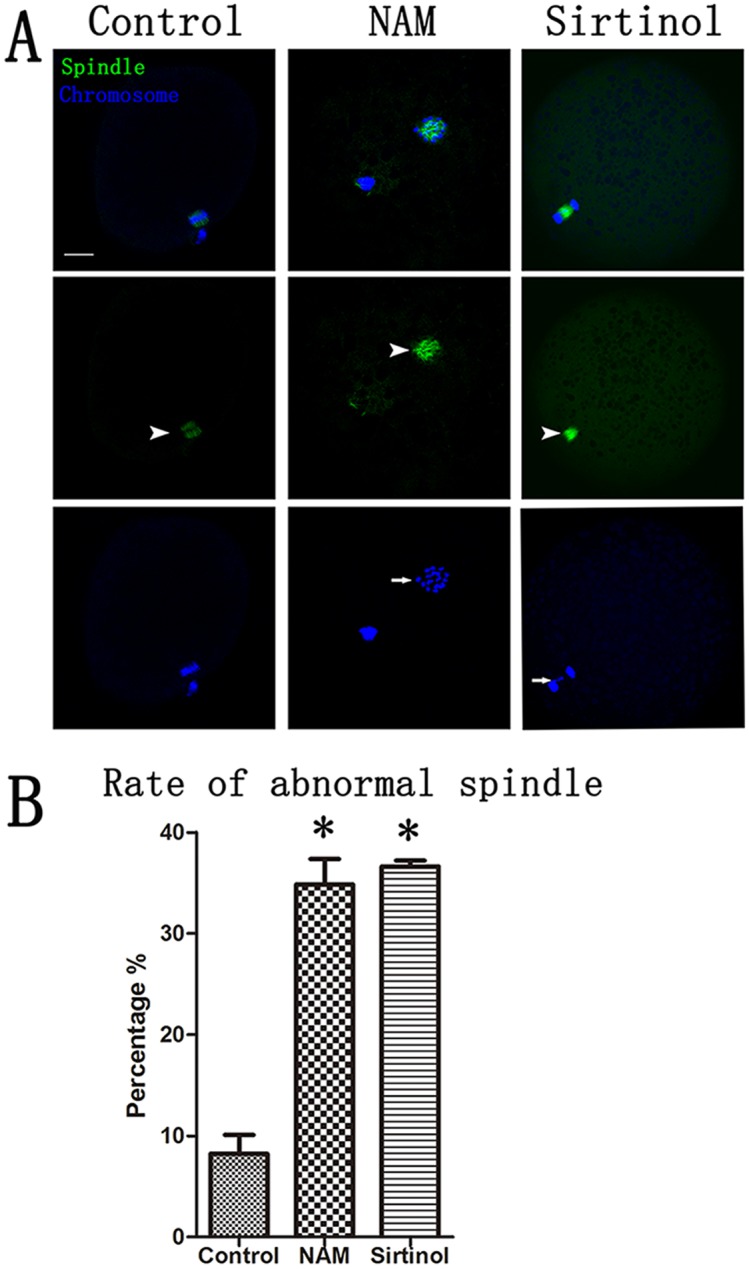
Treatment with NAM and Sirtinol induces meiotic defects in porcine oocytes. (A) Meiotic spindle morphology in second meiosis (MII) oocytes. Control oocytes had barrel-shaped spindles and well aligned chromosomes, whereas spindle defects were frequently observed in NAM and Sirtinol-treated oocytes. Moreover, chromosomes displayed misalignment in treatment oocytes. Green, Tubulin; blue, chromatin. Bar = 20μm (B) Quantification of spindle defects and chromosome misalignment in control and inhibitor-treated oocytes. The percentage of abnormal spindle morphology was significant higher in the NAM- and Sirtinol-treated oocytes than in the control oocytes. *, significant, *p* < 0.05.

To understand why inhibition of Sirtuin activity prevented polar body extrusion, spindle microtubules and chromosomes were labeled, and their organization was analyzed. As shown in Fig 4A, the spindles of control oocytes exhibited normal barrel-shaped morphology, and the chromosomes were well aligned at the metaphase equator. However, in the treatment groups, the MII oocytes exhibited multipolar spindles with randomly scattered chromosomes. In addition, in some inhibitors-treated oocytes, chromosomes were located at both poles of the spindle, with many lagging chromosomes. The percentage of abnormal spindles in the NAM and Sirtinol-treated groups (31.0 ± 7.8%, n = 31; 36.6 ± 0.6%, n = 42, respectively) were significantly higher than that in the control group (8.2 ± 1.9%, n = 46, *p* < 0.05; Fig 4B) (“n” is the average number of each experiment).

## Discussion

The studies presented here were designed to investigate the roles of Sirtuins on porcine oocyte meiotic maturation by using inhibitors NAM and Sirtinol, which block Sirtuin activity. The results indicated that NAM and Sirtinol treatment reduced the rate of polar body extrusionand disrupted actin assembly in porcine oocytes. Moreover, inhibition of Sirtuin activity led to spindle defects and absence of CGFD. The study provides the evidence that Sirtuins are involved in meiotic maturation in porcine oocytes.

Recent studies have shown that Sirtuins play a significant role in preimplantation of porcine embryos, and inhibition of Sirtuins *in vitro* affects embryonic development after parthenogenetic activation and *in vitro* fertilization [22]. In particular, Sirtuins are highly expressed in porcine oocytes [22]. These data indicated that Sirtuins might be involved in meiotic maturation in porcine oocytes. Therefore, we first examined whether inhibition of Sirtuin activity affects cumulus expansion and porcine oocyte maturation. Complete expansion of cumulus is important for oocyte maturation. During ovulation, cumulus cells surrounding the oocyte expand, which in turn regulates meiosis and supports cytoplasmic maturation of oocytes [24]. Furthermore, polar body extrusion is an important criterion for oocyte maturation. After NAM and Sirtinol treatment, the rate of oocyte maturation reduced, as evidenced by the absence of cumulus expansion and failure of oocyte polar body extrusion. In our previous studies, we have demonstrated that deficiency of Sirt2, a member of Sirtuin family, in mouse oocytes affects oocyte maturation because of hyperacetylation of H4K16 and α-Tubulin [23]. These results were consistent with previous reports that NAM suppressed oocyte maturation and inhibited embryo development in X*enopus laevis* [21, 25]. In addition, Resveratrol, an activator of Sirtuins, has been reported to have beneficial effects on oocyte maturation in different animal species [26–29].

Next, we analyzed the expression of actin and formation of CGFD after treatment with NAM and Sirtinol. Oocyte polarity, as well as actin assembly and cortical reorganization [30, 31], are required for polar body extrusion. Spindle migration is a consequence of actin flow, and cortical reorganization is promoted by CGFD [32]. The actin cap and CGFD were absent in NAM/Sirtinol-treated oocytes, while actin intensity on the membrane was markedly decreased. These results indicated that Sirtuins probably affect polar body extrusion by disrupting oocyte polarity. Similar phenotypes were reported upon Arp2/3 and Rho-associated protein kinase (ROCK) inhibition [33, 34], suggesting a relationship between Sirtuins and these proteins. However, additional studies are required to validate this connection. Finally, we observed the elevated percentage of abnormal spindles and chromosome misalignment after treatment with NAM and Sirtinol, suggesting that Sirtuins negatively affect microtubule assembly. In conclusion, our results indicate that the Sirtuins perhaps regulate meiotic maturation in porcine oocytes by facilitating cortical polarity and spindle organzation.
